# P53-dependent upregulation of neutral sphingomyelinase-2: role in doxorubicin-induced growth arrest

**DOI:** 10.1038/cddis.2015.268

**Published:** 2015-10-29

**Authors:** A A Shamseddine, C J Clarke, B Carroll, M V Airola, S Mohammed, A Rella, L M Obeid, Y A Hannun

**Affiliations:** 1 Department of Medicine, Stony Brook University, Health Science Center, Stony Brook, NY 11794-8430, USA; 2 Stony Brook University Cancer Center, Stony Brook, NY 11794-8430, USA; 3 Department of Microbiology and Immunology at Stony Brook University, Stony Brook, NY 11794-8430, USA; 4 The Northport Veterans Affairs Hospital, Northport, NY 11768, USA

## Abstract

Neutral sphingomyelinase-2 (nSMase2) is a ceramide-generating enzyme that has been implicated in growth arrest, apoptosis and exosome secretion. Although previous studies have reported transcriptional upregulation of nSMase2 in response to daunorubicin, through Sp1 and Sp3 transcription factors, the role of the DNA damage pathway in regulating nSMase2 remains unclear. In this study, we show that doxorubicin induces a dose-dependent induction of nSMase2 mRNA and protein with concomitant increases in nSMase activity and ceramide levels. Upregulation of nSMase2 was dependent on ATR, Chk1 and p53, thus placing it downstream of the DNA damage pathway. Moreover, overexpression of p53 was sufficient to transcriptionally induce nSMase2, without the need for DNA damage. DNA-binding mutants as well as acetylation mutants of p53 were unable to induce nSMase2, suggesting a role of nSMase2 in growth arrest. Moreover, knockdown of nSMase2 prevented doxorubicin-induced growth arrest. Finally, p53-induced nSMase2 upregulation appears to occur via a novel transcription start site upstream of exon 3. These results identify nSMase2 as a novel p53 target gene, regulated by the DNA damage pathway to induce cell growth arrest.

Ceramide is a bioactive sphingolipid that has been implicated in numerous biological processes, such as cell cycle arrest and cell death.^1^ Ceramide can be generated through *de novo* synthesis, salvage of sphingosine, or hydrolysis of sphingomyelin.^2^ The latter reaction involves the action of sphingomyelinases, a class of enzymes that differ in their cellular localization and pH optima for activity.^3, 4^ Neutral sphingomyelinase-2 (nSMase2) is the most studied of the nSMases, and nSMase2 activation has been implicated in growth arrest, apoptosis and exosome secretion.^5^

Activation of nSMase2 occurs through different mechanisms. *In vitro* nSMase2 activity is regulated by anionic phospholipids via binding to positively charged sites on the N terminus of the protein.^6, 7, 8^ Post-translational activation occurs also via phosphorylation on five serine residues in response to oxidative stress through a p38 mitogen-activated protein kinase mechanism.^9, 10^ Recently, transcriptional upregulation of nSMase2 became appreciated as a mechanism of enzyme activation. As such, upregulation of nSMase2 was shown to be mediated by Sp1 and Sp3 through direct binding to the nSMase2 promoter in response to all-*trans* retinoic acid (ATRA) and daunorubicin.^11, 12^ Moreover, Runx2 was shown to regulate nSMase2 transcriptionally in response to bone morphogenic protein-2.^13^

Chemotherapeutics are potent generators of ceramide.^14^ The anthracyclin doxorubicin, a daunorubicin analogue, is used as a first-line chemotherapeutic agent for the adjuvant treatment of many tumors such as breast and lung neoplasms.^15, 16^ Multiple mechanisms of action of doxorubicin have been elucidated, but its major antitumor activity is thought to occur through the generation of DNA breaks in the tumor cell. Specifically, doxorubicin binds topoisomerases and stabilizes their interaction with DNA, which prevents the rejoining of nicked DNA following relaxation of supercoils to create DNA breaks.^17^

Following DNA breaks, the DNA damage response is the canonical pathway activated in cells and serves as a protective mechanism to halt cell growth and repair damage.^18, 19^ Mechanistically, the activation is thought to occur in response to both single- and double-strand breaks. The different stimuli lead to the activation of different effector kinases (ATR and Chk1 for ssDNA breaks, ATM and Chk2 for dsDNA breaks).^20, 21^ The signaling cascade converges on p53, which acts as a central hub in integrating signals and regulating effector biologies.^22^ Mutations of p53 occur most commonly in its DNA-binding site and affect its transcriptional activity. As such, these mutations are associated with tumorigenesis, as well as worse outcomes of existing neoplasms.^23^

Ceramide generation in response to anthracyclines has been implicated in mediating antitumor effects such as cell death and growth arrest.^24, 25, 26^ However, the precise mechanism of generation of ceramide, as well as its regulation, remains ambiguous. Moreover, ceramide generation in response to p53 activation has been studied, yet contradicting reports have emerged. Although it has been reported that ceramide is an upstream regulator of p53,^27, 28, 29^ other evidence suggests that p53 regulates ceramide generation in response to specific stresses.^30, 31, 32^ Understanding the regulation of ceramide generation would provide better understanding of tumor responsiveness to doxorubicin. Recently, the effect of daunorubicin on nSMase2 has been described. Daunorubicin activates nSMase2, concomitant with an increase in ceramide formation.^11^

In this study, we first investigate whether doxorubicin, a daunorubicin analogue that is clinically relevant in breast cancer therapy, leads to nSMase2 upregulation and increased ceramide generation in MCF7 breast cancer cells and uncovers the specific mechanism of nSMase2 transcriptional regulation in response to doxorubicin. The data demonstrate that nSMase2 is a downstream target of p53 and is more selectively regulated by ATR and Chk1. Notably, nSMase2 transcriptional activation occurs via a novel transcription start site (TSS). Taken together, these studies identify nSMase2 as an important component of the DNA damage pathway.

## Results

### nSMase2 is induced by doxorubicin in MCF7 cells

**Figure 1:**
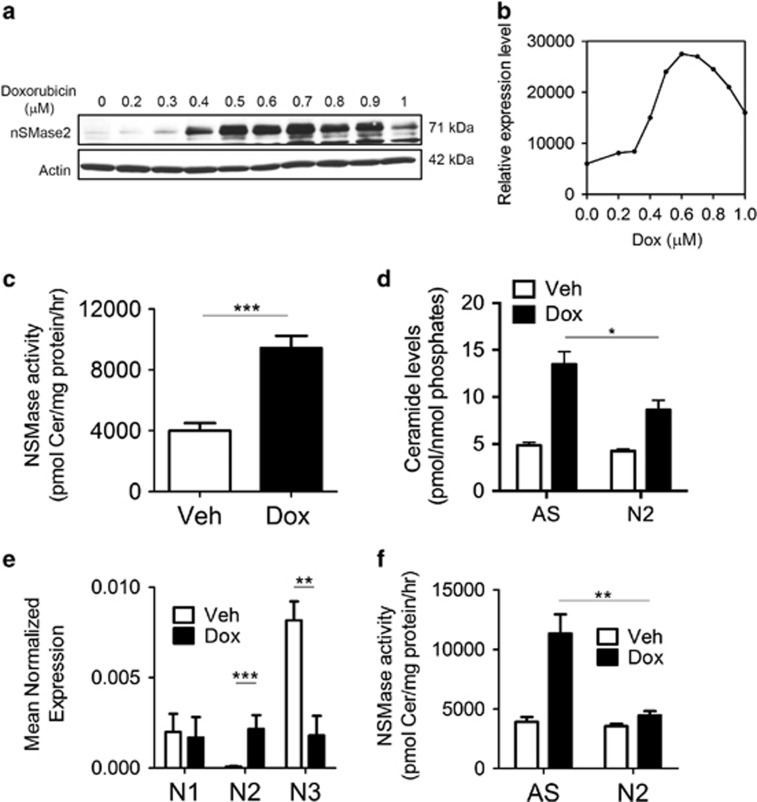
Doxorubicin induces nSMase2 upregulation and ceramide increase in MCF7 cells. (**a**) MCF7 cells were plated in 60 mm dishes and treated with either dimethyl sulfoxide (DMSO) or doxorubicin at different doses for 24 h. Cells were collected, lysed and immunoblotted as described under ‘Materials and Methods'. (**b**) Quantification of nSMase2 induction by ImageJ (NIH, Bethesda, MD, USA) and normalization to actin. (**c**) MCF7 cells were plated in 60 mm dishes and treated for 24 h with either DMSO or doxorubicin. Cells were collected and *in vitro* NSMase assay was performed as described under ‘Materials and Methods', ***P<0.001. (**d**) MCF7 cells were seeded in 60 mm dishes and small interfering RNA (siRNA) was performed to AS (AllStars Negative Control) or nSMase2 (N2) for 24 h, after which either vehicle or doxorubicin were administered and cells were collected 24 h after treatment and analyzed for sphingolipids by liquid chromatography-mass spectrometry (LC/MS) mass spectrometry, *P<0.01. (**e**) MCF7 cells were lysed 24 h after treatment. RNA was isolated and transformed to cDNA to be quantified by quantitative real-time PCR (qRT-PCR) using primers for nSMase1, nSMase2 and nSMase3 as described, **P<0.01 and ***P<0.001. (**f**) MCF7 cells were plated in 60 mm dishes. After 24 h, siRNA to AS or nSMase2 (N2) was transfected for 24 h, after which either vehicle or doxorubicin were added. Cells were collected after 24 h, lysed and *in vitro* sphingomyelinase assay was performed, **P<0.01

Upregulation of nSMase2 was reported in response to daunorubicin, in MCF7 breast cancer cells.^11^ However, as daunorubicin is not used in breast cancer therapy, we sought to study NSMase regulation and ceramide generation using doxorubicin as a clinically relevant chemotherapeutic drug in breast cancer. Doxorubicin treatment of MCF7 cells increased nSMase2 protein levels in a dose- (Figures 1a and b) and time-dependent manner (data not shown), with maximal nSMase2 induction occurring with 600 nM doxorubicin at 24 h. This was concomitant with an increase in total *in vitro* nSMase activity (Figure 1c) and total cellular ceramide levels (Figure 1d). No changes in total sphingomyelin level were detected, possibly owing to the fact that only a small fraction of sphingomyelin needs to be hydrolyzed (<10%) to account for the generated ceramide (Supplementary Figure S1A). Analysis of ceramide according to chain length revealed that C14 and C16 are the major ceramide species upregulated in response to doxorubicin treatment (Supplementary Figure S1B), whereas sphingosine and sphingosine-1-phosphate (Supplementary Figure S1C) levels also increased. The increase in the latter was not statistically significant and independent of p53 (Supplementary Figure S1D). To evaluate the upregulation of other nSMases, mRNA levels of all three cloned human nSMases were analyzed by quantitative real-time PCR. This revealed that nSMase2 was the only N-SMase enzyme induced transcriptionally and was upregulated by around 60-fold (Figure 1e). At higher doses of doxorubicin, nSMase2 was not induced anymore (Supplementary Figure S2). Importantly, knockdown of nSMase2 by siRNA abolished the doxorubicin-mediated increase in total NSMase activity (Figure 1f), as well as decreased the amount of ceramide generated following doxorubicin treatment (Figure 1d). Thus, taken together, these results demonstrate specific induction of nSMase2 by doxorubicin in conjunction with an increase in ceramide levels.

### nSMase2 is upregulated transcriptionally via a novel TSS

**Figure 2:**
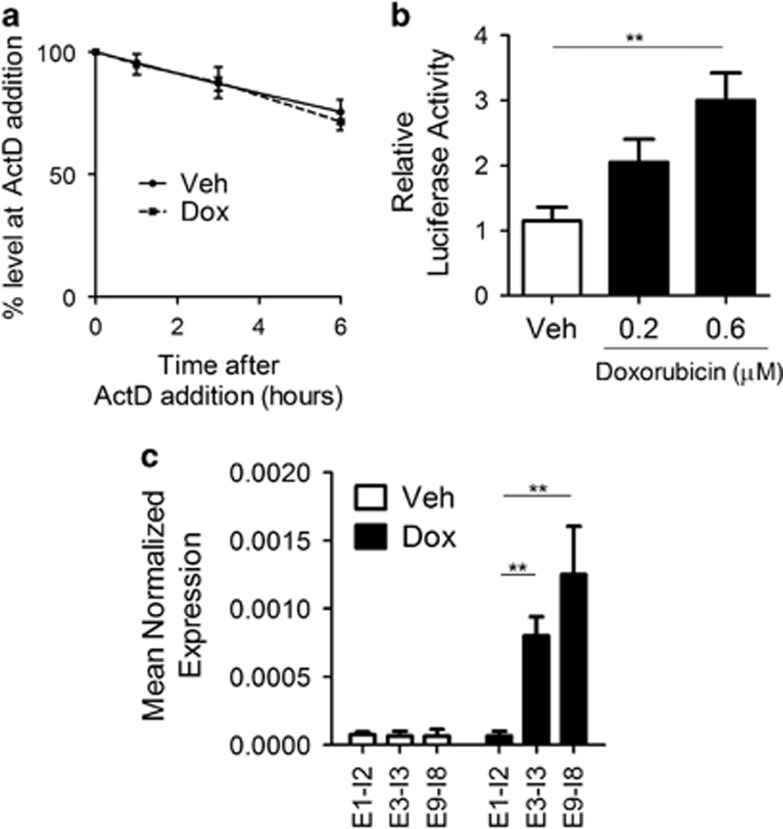
nSMase2 is upregulated transcriptionally via a novel TSS. (**a**) MCF7 cells were seeded in 60 mm dishes and treated with vehicle or Doxorubicin for 24 h. After that, 10 nM of actinomycin D was added for 1, 3 and 6 h. Cells were lysed, RNA was isolated and transformed to cDNA and quantitative real-time PCR (qRT-PCR) was performed with nSMase2 primers. (**b**) MCF7 cells were seeded in 60 mm dishes and transfected with LacZ and pGL3 basic containing the first 1600 bp upstream of exon for 24 h, after which cells were treated with doxorubicin. Cells were then collected and both luciferase and *β-*galactosidase assays were performed as described under ‘Materials and Methods', **P<0.01. (**c**) Representation of exonic sequences of nSMase2. Cells were treated with vehicle and doxorubicin for 24 h, after which cells were collected, RNA was isolated and transformed to cDNA. qRT-PCR was performed using exonic intronic primers, exon1–intron2 (E1–I2), exon3–intron3 (E3–I3) and exon9–intron8 (E9–I8), **P<0.01

As the levels of nSMase2 mRNA increased, it was prudent to determine if the effect was purely transcriptional. To rule out the effects of doxorubicin on mRNA stability, cells were treated with doxorubicin for 24 h, after which actinomycin D was added. As seen in Figure 2a, nSMase2 mRNA stability was similar in both vehicle- and doxorubicin-treated cells. To determine if the effect is through promoter activation of nSMase2, the putative promoter of nSMase2, encompassing the first 1000 base pairs upstream of exon 1, was cloned, and its activity was evaluated by luciferase reporter assays. Doxorubicin-treated MCF7 cells displayed a 2.5-fold increase in luciferase activity, which suggested activation of the putative promoter (Figure 2b). However, as the fold change in promoter activity and mRNA levels did not match (2.5-fold *versus* 60-fold), it was important to investigate the presence of an alternative promoter. The annotated nSMase2 5′-untranslated region (UTR) encompasses exons 1 and 2, as well as part of exon 3. Using intronic–exonic primers, heteronuclear RNA (unspliced mRNA) of nSMase2 was amplified. The results suggested that the 5′-UTR of nSMase2 mRNA, upregulated after doxorubicin treatment, does not include exons 1 and 2 as exon1–intron2 primers failed to amplify to the same extent as downstream exonic–intronic junctions (Figure 2c). These results point to a doxorubicin-specific transcriptional regulation of nSMase2 through a novel TSS upstream of exon 3, but not including exons 1 and 2.

### nSMase2 transcriptional activation is independent of known transcriptional regulators

**Figure 3:**
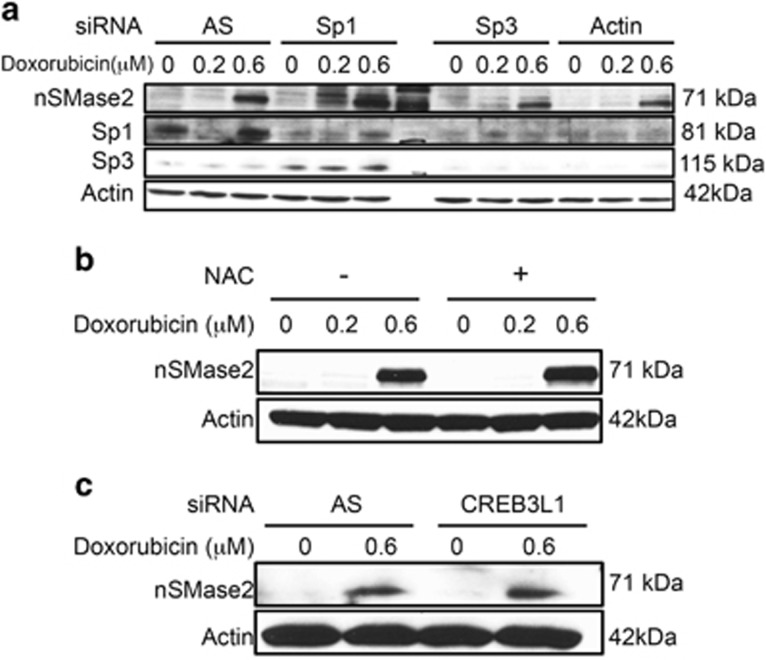
nSMase2 upregulation is independent of known transcriptional regulators of nSMase2. (**a**) MCF7 cells were seeded in 60 mm dishes and transfected with siRNA to AllStars Negative Control (AS), Sp1, Sp3 or both together. After 24 h, cells were treated with vehicle, and 0.2 or 0.6 *μ*M doxorubicin. After 24 h, cells were collected and immunoblotted for nSMase2, Sp1, Sp3 and actin. (**b**) MCF7 cells were seeded in 60 mm dishes. One hour before stimulation with doxorubicin or vehicle, they were pretreated with *N*-acetylcysteine (NAC). Cells were collected and immunoblotted for nSMase2 and actin. (**c**) MCF7 cells were seeded in 60 mm dishes and transfected with siRNA to AS or CREB3L1. After 24 h, they were treated with vehicle or 0.6 *μ*M doxorubicin. Cells were collected and immunoblotted for nSMase2 and actin

Transcriptional regulation of nSMase2 has been described in response to different stimuli. Sp1 and Sp3 were shown to regulate nSMase2 transcriptionally in response to daunorubicin and ATRA.^11, 12^ Formation of reactive oxygen species (ROS) was shown to modulate nSMase2, and doxorubicin is a potent generator of ROS.^32, 33, 34^ In addition, Denard *et al.* described regulation of ceramide production by CREB3L1 following doxorubicin in MCF7 cells.^35^ As such, it was next essential to determine the effect of these transcriptional regulators on nSMase2 in response to doxorubicin. As can be seen, siRNA knockdown of Sp1 and Sp3 did not prevent nSMase2 upregulation after doxorubicin treatment (Figure 3a). Moreover, pre-treatment with *N*-acetylcysteine, a quencher of ROS, had no effect of nSMase2 induction (Figure 3b). Finally, knockdown of CREB3L1 by siRNA did not inhibit nSMase2 upregulation in response to doxorubicin (Figure 3c). CREB3L1 knockdown was verified by qRT-PCR (Supplementary Figure S3). Taken together, these results suggest that nSMase2 transcriptional activation is independent of known regulators, and is possibly due to a new previously undescribed mechanism.

### nSMase2 is regulated transcriptionally by DNA-specific components of the DNA damage pathway

**Figure 4:**
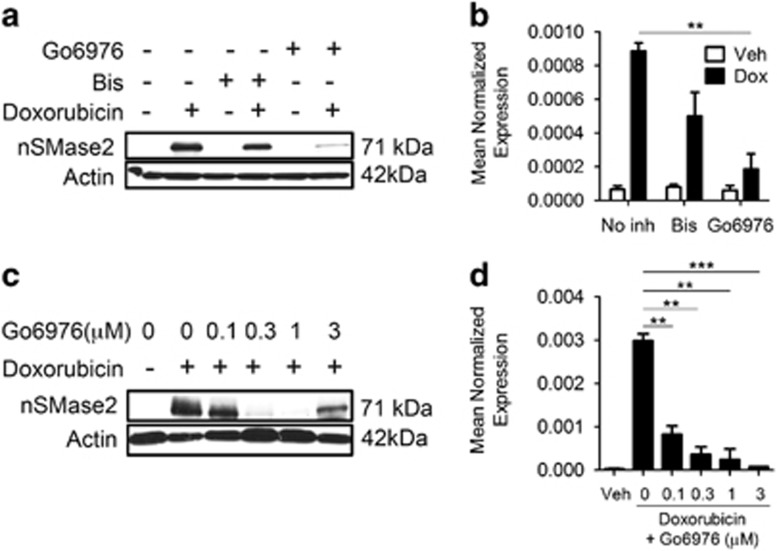
Go6976 regulates nSMase2 transcriptionally. (**a**–**d**) MCF7 cells were seeded in 60 mm dishes and, 1 h before stimulation with either vehicle or doxorubicin, were pretreated with the specified inhibitor, and 24 h after treatment, cells were collected and immunoblotted for nSMase2 and actin (**a** and **c**), or RNA was isolated and transformed to cDNA and quantitative real-time PCR (qRT-PCR) was performed for nSMase2 (**b** and **d**), **P<0.01 ***P<0.001

Ito *et al.*^12^ described an effect of Bis (a classical and novel PKC inhibitor) on nSMase2 in response to ATRA. In our study, pretreatment with Go6976 (a classical PKC inhibitor) exerted a robust inhibitory effect on nSMase2 induction, whereas Bis pretreatment had a mild effect (Figures 4a and b). These results were difficult to reconcile as Bis should also inhibit the classical PKCs and suggested an off-target effect of Go6976. Typically, Go6976 is used as a PKC inhibitor at concentrations that range between 1 and 3 *μ*M. To evaluate the optimal concentration for the effects of Go6976, cells were pretreated with different concentrations of Go6976 and nSMase2 induction was evaluated. Unexpectedly, inhibition of nSMase2 induction occurred at concentrations as low as 300 nM at the protein level (Figure 4c) and 100 nM at the mRNA level (Figure 4d). These results suggested that PKCs are not involved in mediating nSMase2 induction.

**Figure 5:**
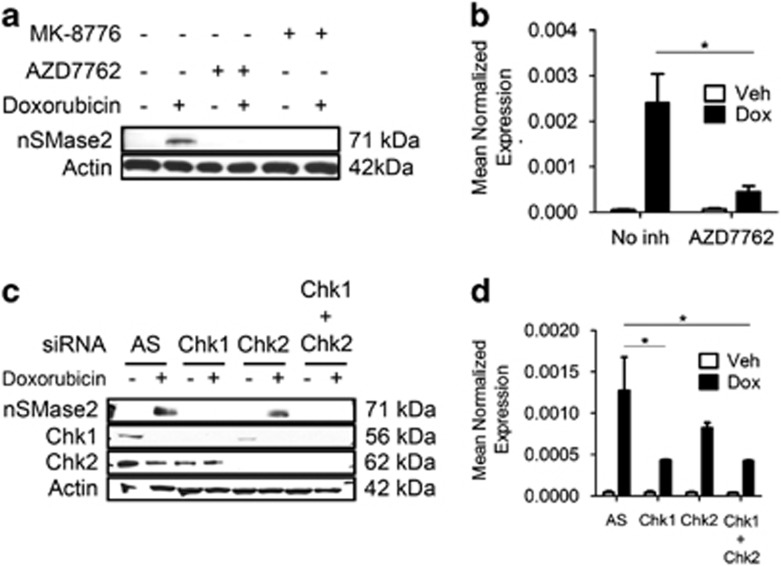
Chk1 regulates nSMase2 transcriptionally. (**a** and **b**) MCF7 cells were seeded in 60 mm dishes and were pretreated with the specified inhibitor 1 h before stimulation with either vehicle or doxorubicin. Twenty-four hours after treatment, cells were collected and immunoblotted for nSMase2 and actin (**a**), or RNA was isolated and transformed to cDNA and quantitative real-time PCR (qRT-PCR) was performed for nSMase2 (**b**), *P<0.05. (**c** and **d**) MCF7 cells were seeded in 60 mm dishes and siRNA was performed using AllStars Negative Control (AS), Chk1, Chk2 or Chk1+Chk2. After 24 h, cells were stimulated with either vehicle or doxorubicin and collected for analysis by immunoblotting (**c**) or qRT-PCR (**d**), *P<0.05

The best characterized off-target of Go6976 is the checkpoint kinase Chk1, which is inhibited by Go6976 at nanomolar concentrations.^36^ Moreover, doxorubicin is a DNA damage agent that activates effector kinases of the DNA damage response.^37^ Therefore, it was rational to hypothesize that nSMase2 transcription may be regulated by Chk1 and other DNA damage regulators. Using the nonspecific Chk1/Chk2 inhibitor AZD7762, as well as the specific Chk1 inhibitor MK-8776, a robust downregulation of nSMase2 induction was observed both at the protein (Figure 5a) and mRNA level (Figure 5b). Individual knockdown of Chk1 and Chk2 revealed that Chk1 is the major isoform responsible for nSMase2 induction in response to doxorubicin at the protein level (Figure 5c), as well as the mRNA level (Figure 5d).

**Figure 6:**
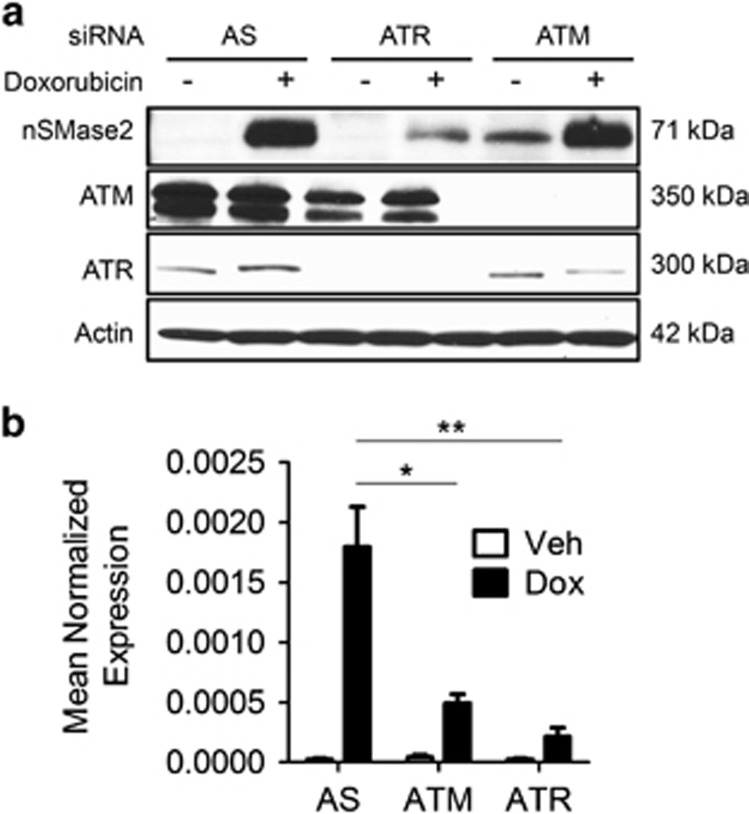
Ataxia Telangiactesia related-protein (ATR) regulates nSMase2 transcriptionally upstream of Chk1. (**a** and **b**) MCF7 cells were seeded in 60 mm dishes, and small interfering RNA (siRNA) knockdown was performed using AS, for 24 h. After that, vehicle or doxorubicin were added and cells were collected and immunoblotted for nSMase2, ataxia telangiectasia mutated (ATM), ATR and actin (**a**), or RNA was isolated and transformed to cDNA and quantitative real-time PCR (qRT-PCR) was performed for nSMase2 (**b**), *P<0.05 and **P<0.01

As Chk1 is an effector kinase of the DNA damage response that is in turn activated by upstream kinases, namely ATM and ATR, we undertook to determine the signaling pathway upstream of Chk1, which controls nSMase2 induction. To determine whether ATM or ATR influence nSMase2 upregulation, individual siRNA knockdown of ATM and ATR was performed. ATR knockdown significantly downregulated nSMase2 induction both at the protein (Figure 6a) and mRNA level (Figure 6b). In contrast, knockdown of ATM had very modest effects (Figure 6a). These results place nSMase2 as a downstream transcriptional target of both ATR and Chk1.

### P53 is both necessary and sufficient for nSMase2 transcriptional upregulation

**Figure 7:**
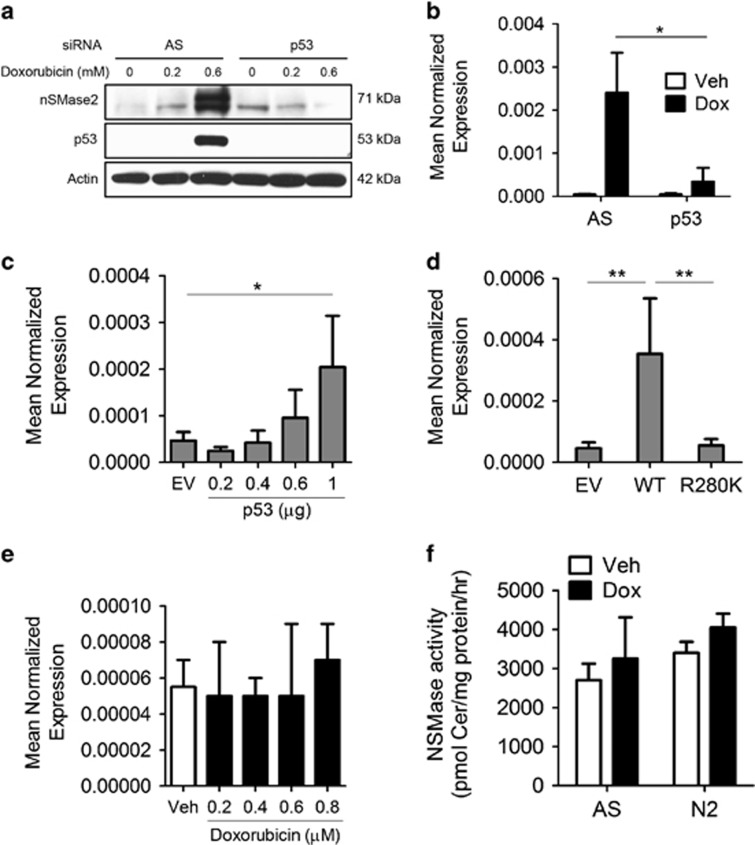
P53 is both necessary and sufficient for the induction of nSMase2. (**a** and **b**) MCF7 cells were seeded in 60 mm dishes and siRNA knockdown was performed using AS or p53 for 24 h. After that, vehicle or doxorubicin were added and cells were collected and immunoblotted for nSMase2, p53 and actin (**a**), or RNA was isolated and transformed to cDNA and quantitative real-time PCR (qRT-PCR) was performed for nSMase2 (**b**), *P<0.05. (**c** and **d**) MCF7 cells were seeded in 60 mm dishes and transfected either with control (pcDNA), increasing concentrations of WT p53 plasmid (**c**), or different p53 mutants (**d**). Cells were collected and RNA was isolated, after which it was transformed to cDNA. qRT-PCT was performed for nSMase2, *P<0.05 and **P<0.01. (**e**) MDA-MB-231 cells were treated with different concentrations of doxorubicin. Cells were collected, RNA was isolated and qRT-PCR performed for nSMase2. (**f**) MDA-MB-231 cells were transfected with AS or nSMase2 siRNA after which they were treated with vehicle or doxorubicin. Cells were collected and *in vitro* NSMase activity assay was performed

The main transcriptional functions of ATR and Chk1 occur through the activation of the tumor suppressor p53. Therefore, it became important to determine whether p53 was required for nSMase2 induction in response to doxorubicin. Knockdown of p53 by siRNA prevented nSMase2 induction in response to doxorubicin both at the protein (Figure 7a) and the mRNA levels (Figure 7b). Reciprocally, overexpression of p53 yielded a dose-dependent increase in nSMase2 expression in the absence of doxorubicin (Figure 7c). To confirm that this is dependent on the transcriptional activity of p53, a p53 construct carrying the common mutation R280K in the DNA-binding domain was used to test for nSMase2 induction upon its overexpression. Overexpression of wild-type (WT) p53 but not mutant R280K p53 resulted in nSMase2 induction in the absence of genotoxic stress (Figure 7d). To validate these results, MDA-MB-231 breast cancer cells, which carry the p53-R280K mutation, were stimulated with doxorubicin. As can be seen, doxorubicin treatment had no effect on nSMase2 mRNA levels (Figure 7e) in these cells. Furthermore, activity assays did not show significant increase in NSMase activity (Figure 7f).

Collectively, these results demonstrate that p53 is both necessary for nSMase2 transcriptional activation in response to doxorubicin and sufficient to upregulate nSMase2 transcriptionally in the absence of genotoxic stress.

### nSMase2 mediates growth arrest in response to doxorubicin

**Figure 8:**
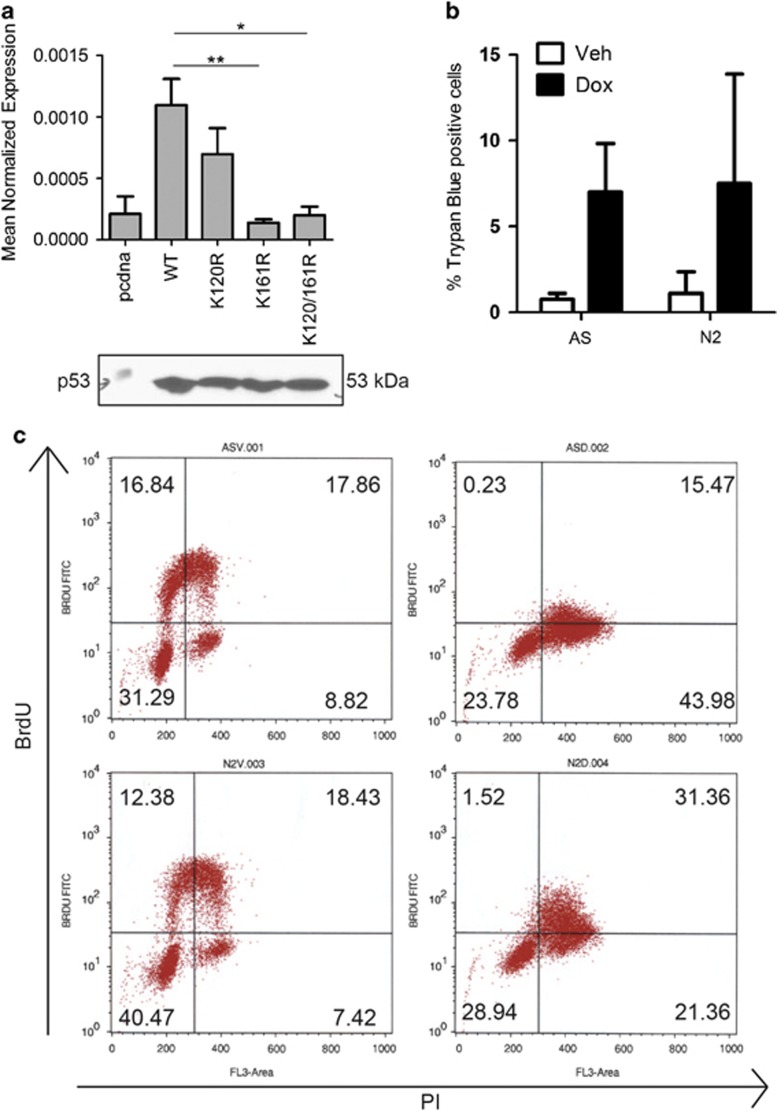
nSMase2 mediates growth arrest in response to doxorubicin. (**a**) MCF7 cells were seeded in 60 mm dishes and transfected either with control (pcDNA), WT p53 plasmid or different p53 mutants, *P<0.05 and **P<0.01. Cells were lysed either for protein extraction or RNA isolation. RNA was reverse transcribed to cDNA, and nSMase2 expression was determined by qRT-PCR. Also, protein extraction was performed and samples were immunoblotted for p53 (**b**), MCF7 cells were seeded in 60 mm dishes and siRNA knockdown was performed using AS or nSMase2 for 24 h. After that, vehicle or doxorubicin were added and cells were collected, stained with Trypan Blue and counted (**c**), MCF7 cells were seeded in 60 mm dishes and siRNA knockdown was performed using AS or nSMase2 for 22 h. A pulse of BrdU was given to cells for 30 min and they were collected and analyzed by flow cytometry

Lysine mutants of p53 have become the subject of increasing interest. Studies have shown that these mutants do not affect the tumor-suppressive functions of p53 in murine models, yet they impair p53-mediated growth arrest and apoptotic functions. Therefore, the ability of these mutants to induce nSMase2 was evaluated through p53 mutant constructs harboring lysine-to-arginine mutations at positions 120, 161 and both 120 and 161. Although the K120R mutant induced nSMase2, the K161R as well as the K120-161R double mutants did not in comparison with WT p53 (Figure 8a). The specific nature of nSMase2 induction in response to WT p53 but not the K161R mutant suggested possible biological functions of nSMase2 upregulation in response to doxorubicin. As such, the involvement of nSMase2 in mediating either apoptotic or growth arrest functions in response to doxorubicin was studied. At the concentrations at which it induces nSMase2, doxorubicin did not induce cell death, and nSMase2 knockdown did not change Trypan blue uptake (Figure 8b) nor did it change annexin V/PI staining of MCF7 cells (Supplementary Figure S4). On the other hand, BrdU analysis revealed that, whereas doxorubicin-treated control cells did not incorporate BrdU, the cells with nSMase2 downregulation had increased uptake of BrDU (Figure 8c). Taken together, these results suggest a role of nSMase2 in mediating growth arrest following doxorubicin treatment.

## Discussion

In this study, we have explored the regulation of nSMase2 and ceramide generation in breast cancer cells treated with doxorobucin. We report that nSMase2 is the primary N-SMase regulated by doxorubicin and that this is independent of reported transcriptional regulators of nSMase2. Instead, nSMase2 induction was strongly dependent on p53, ATR and Chk1, and potentially occurs through an alternate transcriptional start site in the nSMase2 gene. Collectively, these data define a novel pathway of nSMase2 regulation by DNA damage effector proteins, and the results shed light on the mechanisms of ceramide generation by doxorubicin in breast cancer.

A number of studies have implicated N-SMase activity in the cellular responses to cytokines and stress including chemotherapeutics.^26, 38, 39, 40, 41, 42^ However, little is known about the specific N-SMase isoforms involved or the mechanisms of their regulation. In the current study, nSMase2 was identified as the major doxorubicin-responsive N-SMase in breast cancer cells. Previously, nSMase3 was shown to be upregulated following doxorubicin treatment acutely to mediate cancer cell sensitivity to the drug.^43^ However, recent data raised doubt on whether nSMase3 functions as a sphingomyelinase.^44^

Notably, nSMase2 induction was transcriptional, as evidenced by effects on hnRNA and lack of effects on mRNA stability, and consistent with a previous study using the doxorubicin analogue, daunorubicin. However, this induction appears to be through a novel regulatory pathway involving the tumor suppressor protein p53, and the DNA damage effectors ATR and Chk1 – as evidenced by both pharmacological inhibitors and siRNA knockdown. Furthermore, p53 overexpression was sufficient to induce nSMase2 transcriptionally, even in the absence of genotoxic stress. These results differ from daunorubicin induction of nSMase2 in MCF7 cells, which was reported to require the transcription factors Sp1 and Sp3 – which appear to be dispensable in our system.^11^ Notably, the previous study relied on pharmacological inhibitors and thus off-target effects could account for the differences observed. The lack of role of Sp1 and Sp3 in the doxorubicin response is also consistent with observations suggesting an alternative TSS for nSMase2. In the previous study, Sp1 and Sp3 were observed to interact with the nSMase2 gene upstream of exon 1. In contrast, doxorubicin does not significantly increase transcription through exon 1 or exon 2 of the *Smpd3* gene as evidenced by both RNA sequence and hnRNA analysis with specific exon–intron primers. Although attempts to fully define this alternate TSS have thus far been unsuccessful, it should be noted that multiple TSS sites are highly common for genes with long first introns as is seen with the *Smpd3* gene (>70 kb).

Importantly, the linking of nSMase2 expression to both ATR and Chk1 suggests that nSMase2 induction by p53 may occur in very specific cellular contexts, rather than functioning as a general effector of DNA damage. Indeed, ATR and Chk1 activation is known to depend on ssDNA breaks generated following genotoxic stress^21^ and doxorubicin (as well as daunorubicin) is able to induce both ssDNA and dsDNA breaks. Notably, UV radiation – another activator of ssRNA breaks, ATR and Chk1 – has also been described to induce N-SMase activity, and tumor resistance to apoptosis by UV has been linked to failure of N-SMase activation.^45, 46^ Although the specific NSMase as well as its regulation had not been studied, our study suggests that nSMase2 could be the isoform also responsible for NSMase activity increase in response to UV. These data would predict that stimuli that activate the ATM-Chk2 axis such as *γ*-irradiation (IR) would not induce nSMase2. Indeed, although IR has been reported to generate ceramide, this was attributed to the activation of acid sphingomyelinase and not N-SMase and was also reported to be p53-independent.^47, 48^ Further to this, a number of studies have reported p53-dependent generation of ceramide in response to different stresses; however, there is a paucity of information on the underlying mechanisms by which p53 performs these functions.^43, 49^ To date, only two studies have focused on the regulation of specific sphingolipid enzymes by p53. The first showed loss of sphingosine kinase 1 following genotoxic stress that may occurr in a p53-dependent manner,^50^ and the second elucidated a mechanism of p53-dependent induction of CerS6 in response to folate stress.^30^ However, this clearly suggests that the pathway of ceramide generation is dependent on the specific stress, the cellular context and, as noted above, activation of additional signaling pathways. Furthermore, the upregulation of WT p53 in response to chemotherapy can mediate cancer cell resistance to these agents through activation of pathways of cell cycle arrest and DNA repair. As ceramide has been proposed to regulate growth arrest in response to p53, a wider understanding of the differential regulation and effects of specific ceramide-generating pathways is of paramount importance. This would allow the targeting of specific enzymes through inhibitors in conjunction with chemotherapy to promote its efficacy. In fact, doxorubicin is administered as a bolus with chemotherapy, and while the initial concentrations in tumors are very high, there is a prolonged exposure to low doses of doxorubicin when the levels decrease and also in poorly perfused tumors. As such, targets that would inhibit the protective mechanism of growth arrest at these concentrations should have beneficial effects on tumor therapy when combined with doxorubicin treatment, and nSMase2 is a target well worth exploring given its effects on the growth arrest functions of doxorubicin.

Finally, the identification of genes that are differentially regulated by WT *versus* mutant p53 is gaining increasing importance with the study of both loss and gain of functions of mutant p53 attempting to identify reasons behind the aggressiveness of cancers with mutations in p53. Given the established roles of ceramide generation in tumor-suppressive biologies such as growth arrest, senescence and apoptosis, as well as anti-invasiveness, one possible hypothesis for the aggressiveness of p53 mutant tumors is its inability to activate or degrade ceramide-metabolizing enzymes. The lack of effect of such p53 mutants on nSMase2 induction would be consistent with such a hypothesis. Consequently, manipulation of the sphingolipid pool in cancer cells by targeting the sphingolipid enzymes might offer a viable therapeutic approach that needs further exploration and clarification.

In conclusion, this study identifies nSMase2 as the major neutral N-SMase upregulated in response to doxorubicin, a first-line agent used in the treatment of breast cancer, and suggests that nSMase2 is a primary pathway of ceramide generation in the doxorubicin response. Moreover, nSMase2 induction is dependent on ATR, Chk1 and p53. Collectively, these results place nSMase2 as an essential part of the DNA damage pathway. Further investigation of the potential roles of nSMase2 in this pathway could provide better understanding of the biological relevance of its activation as well as potential benefits of targeting nSMase2 in conjunction with chemotherapy.

## Materials and Methods

### Materials

MCF7 breast carcinoma cells were obtained from ATCC (Manassas, VA, USA). RPMI culture medium, fetal bovine serum and SuperScript reverse transcriptase were obtained from Invitrogen (Carisbad, CA, USA). Antibodies for nSMase2 (H195), Sp1 (PEP2) and Sp3 (D20) were purchased from Santa Cruz Biotechnology (Santa Cruz, CA, USA). Antibodies for Chk1 (2360), Chk2 (2662), ATM (2873), ATR (2790), p53 (9282) and actin (4967) were from Cell Signaling Technologies (Beverly, MA, USA). The inhibitors AZD7762 (S1532) and MK-8776 (S2735) were from Selleck Chem (Boston, MA, USA). Go6976 (2253) was purchased from Tocris Biosciences (Bristol, UK). Bisindolylmaleimide I (13298) was obtained from Cayman Chemicals (Ann Arbor, MI, USA). siRNA for TP53 (s607) and CREB3L1 (s40546) were from Life Technologies (Grand Island, NY, USA). All other siRNAs, CHEK1 (SI00024570), CHEK2 (SI00095305), ATM (SI00000840), ATR (SI00023107), Sp1 (SI150983) and Sp3 (SI0004788) were from Qiagen (Hiden, Germany). Porcine brain sphingomyelin and phosphatidylserine were from Avanti Polar Lipids (Alabaster, AL, USA). Doxorubicin, actinomycin D and, unless indicated otherwise, all other chemicals were obtained from Sigma (St. Louis, MO, USA).

### Cell culture and siRNA

MCF7 cells were grown at 37** **°C with 5% CO_2_ in 10% fetal bovine serum in RPMI. Cells were subcultured in 60 mm dishes (175 000 cells) and the medium was changed 1 h before the start of experiments. For siRNA experiments, cells were plated in 60 mm dishes (100 000 cells), and 24 h later, they were transfected with 10 nM negative control or 10 nM of the desired siRNA using Lipofectamine RNAiMAx reagent (Life Technologies) according to the manufacturer's protocol. After 24 h, cells were incubated in the fresh medium for 1 h before treatment.

### Construction of p53 mutants and cellular overexpression

The plasmid pcDNA-WTp53 was a generous gift from Dr. Ute Moll. The R280K, K120R and K161R mutants were produced using QuikChange Site-directed Mutagenesis (Agilent Technologies, Santa Clara, CA, USA). The primers used for the mutants are: R280K (F) 5′-TGTGCCTGTCCTGGGAAAGACCGGCGCACAGAG-3′ and (R) 5′-CTCTGTGCGCCGGTCTTTCCCAGGACAGGCACA-3′ K120R (F) 5′-GCATTCTGGGACAGCCAGGTCTGTGACTTGCACGTACTCCCCTGC-3′ and (R) 5′-GCAGGGGAGTACGTGCAAGTCACAGACCTGGCTGTCCCAGAATGC-3′ K161R (F) 5′-GCACGTACTCCCCTGCCCTCAACAGGATGTTTTGCCAACTGGC-3′ and (R) 5′-GCCAGTTGGCAAAACATCCTGTTGAGGGCAGGGGAGTACGTGC-3′.

For overexpression of vectors in mammalian cells, MCF7 cells were subcultured in 60 mm dishes (350 000 cells) and the medium was changed 1 h before the experiment and then were transfected with 1 *μ*M of the plasmid containing the insert of interest or its complementary empty vector using Xtreme gene transfection reagent (Roche, Basel, Switzerland) according to the manufacture's protocol. Cells were collected 24 h later and further experiments were conducted.

### Protein extraction and immunoblotting

For cellular protein extraction, cells were scraped in 0.75% SDS and lysed by sonication. Bradford reagent (Bio-Rad, Hercules, CA, USA) was used to determine protein concentration before immunoblotting. Lysates were mixed with equal volumes of 2x Laemelli buffer (Bio-Rad) and boiled for 5 min. The protein was separated by SDS-PAGE using the Criterion gels (Bio-Rad) and immunoblotted as described previously.^51^

### Real-time PCR

RNA extraction was performed using the Purelink RNA Kit (Life Technologies) according to the manufacturer's protocol. RNA quality and concentration was verified by nanodrop, after which 1 *μ*g of RNA was transformed into cDNA using the Quanta cDNA Kit (Gaithersburg, MD, USA) according to the manufacturer's protocol. For qRT-PCR, reactions were run in triplicates in 96-well plates with each reaction containing 10 *μ*l of 2 × iTAQ mastermix, 5 *μ*l of cDNA, 1 *μ*l of Taqman primer probe and 4 *μ*l of water. The primer probes used were purchased from Life Technologies and amplified with nSMase2 (cat. no. 4331182), actin, (cat. no. 4448484) and CREB3L1 (cat no. 4331182).

### Neutral sphingomyelinase assay

Neutral sphingomyelinase activity was assayed as described previously using ^14^C-[methyl]sphingomyelin as a substrate.^51^ Briefly, SM from bovine brain (Avanti polar lipids) and C14 PS (Avanti polar lipids) were dried under N_2_ (g) and resuspended in Triton X-100 mixed micelles by sonication. The final reaction conditions contained 0.1% Triton X-100, 100 mM Tris buffer, pH 7.5, 20 mM MgCl_2_ and 5 mM DTT with 5 mol% SM (SM only) or 5 mol% SM+5 mol% PS (SM+PS). Cellular lysates containing 80 *μ*g of protein were added to 100 *μ*l of assay buffer and were incubated for an hour. After that, reaction was quenched and a modified Bligh and Dyer extraction was performed. The aqueous phase (700 *μ*l of supernatant) was transferred to scintillation vials containing 3 ml of scintillation fluid and counted.

### Analysis of cellular sphingolipids

Before collection, cells were incubated in serum-free medium (RPMI with 0.1% fatty-acid free BSA) for 3 h. Cells were scraped and pelleted and extraction and analysis by LC/MS mass spectrometry was performed as described previously.^51^ Lipids were normalized to total phosphate levels of selected sample.

### Promoter cloning and luciferase assays

Using human genomic DNA (Roche), we amplified 1500 bp upstream of exon 1 using the following primers 5′-CGGCTCGAGGGAGGTGTATGTGAATGAGGTTCC-3′ and 5′-CCCAAGCTTGGGTCCGGAGCCTCCCTCAGACTC-3′. The insert was purified and cloned into a pGL3 basic vector (Life Technologies). Cells were transfected with equal plasmid amount of pGL3 containing insert and a control plasmid containing *β*-galactosidase. After 24 h, cells were treated with doxorubicin and then assayed for promoter activation using a Luciferase Assay Kit (Promega, Madison, WI, USA). Results were normalized to *β*-galactosidase activity as measured by a *β*-galactosidase assay (Agilent).

### Statistical analysis

All experiments are n of 3 unless expressed otherwise. Bars are representative of means and standard deviation. Two-way ANOVA was used for the analysis of samples with multiple variables (for example treatment and siRNA) and one-way ANOVA was used for the rest. Significance is achieved at *P*<0.05.

## Abbreviations

nSMase2: neutral sphingomyelinase-2
ATRA: all-*trans* retinoic acid
TSS: transcription start site
ActD: actinomycin D
UTR: untranslated region
ROS: reactive oxygen species
NAC: *N*-acetylcysteine
BrdU: bromodeoxyuridine
hnRNA: heteronuclear RNA
Veh: vehicle
Dox: doxorubicin
AS: AllStars Negative Control siRNA
