# Supplementary material for: P53-dependent upregulation of neutral sphingomyelinase-2: role in doxorubicin-induced growth arrest
Source: Cell Death Dis. 2015 Oct 29;6(10):e1947–. doi: 10.1038/cddis.2015.268 (PMC4632297; doi:10.1038/cddis.2015.268)
Supplement: Supplementary Figures [file cddis2015268x1.pdf]

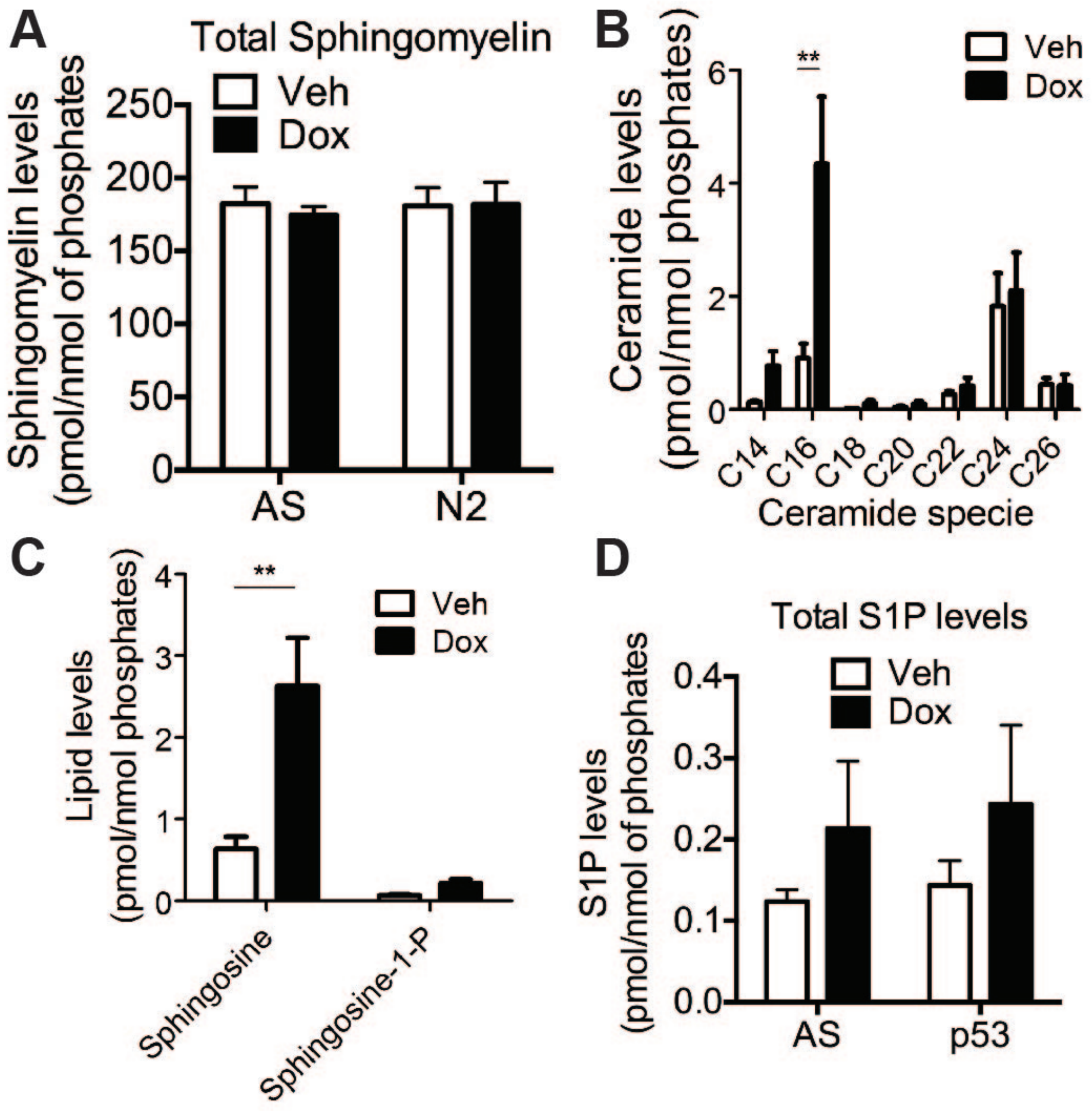

SUPPLEMENTARY FIGURE 1: Sphingolipid levels following Doxorubicin treatment

MCF7 cells were collected 24 hours after treatment and analyzed for sphingolipids by LC/MS mass spectrometry. Shown are total sphingomyelin levels All Star negative control siRNA (AS) or nSMase2 siRNA (N2) (A), ceramide species levels (B), Sphingosine levels and Sphingosine-1-phosphate levels (C), and S1P levels following

treatment with doxorubicin and knockdown with either AS or p53 siRNA $*p<0.05$ $**p<0.01$ $****p<0.0001$

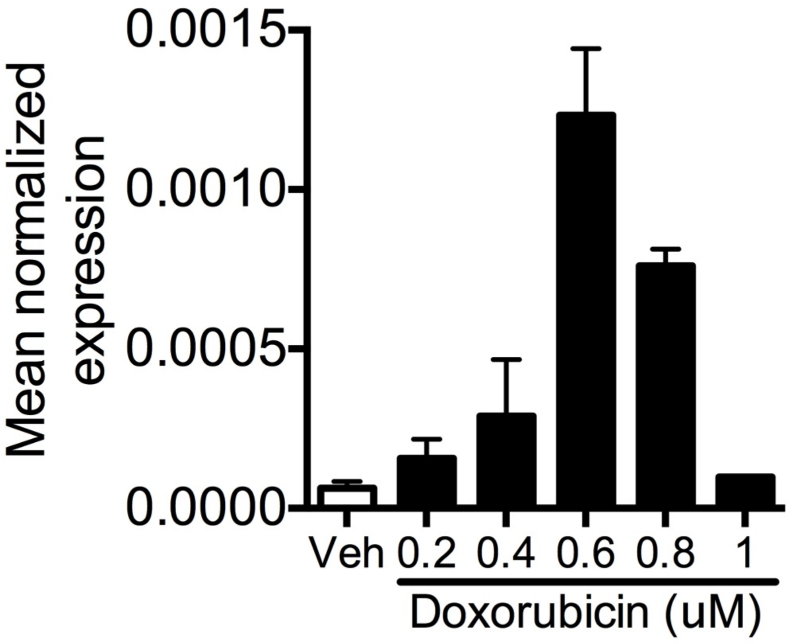

SUPPLEMETARY FIGURE 2: nSMase2 is induced transcriptionally in a dose-dependent manner

MCF7 cells were seeded in 60 mm dishes after which vehicle or different concentrations of doxorubicin were added, cells were collected, RNA was isolated and transformed to cDNA and qRT-PCR was performed for nSMase2.

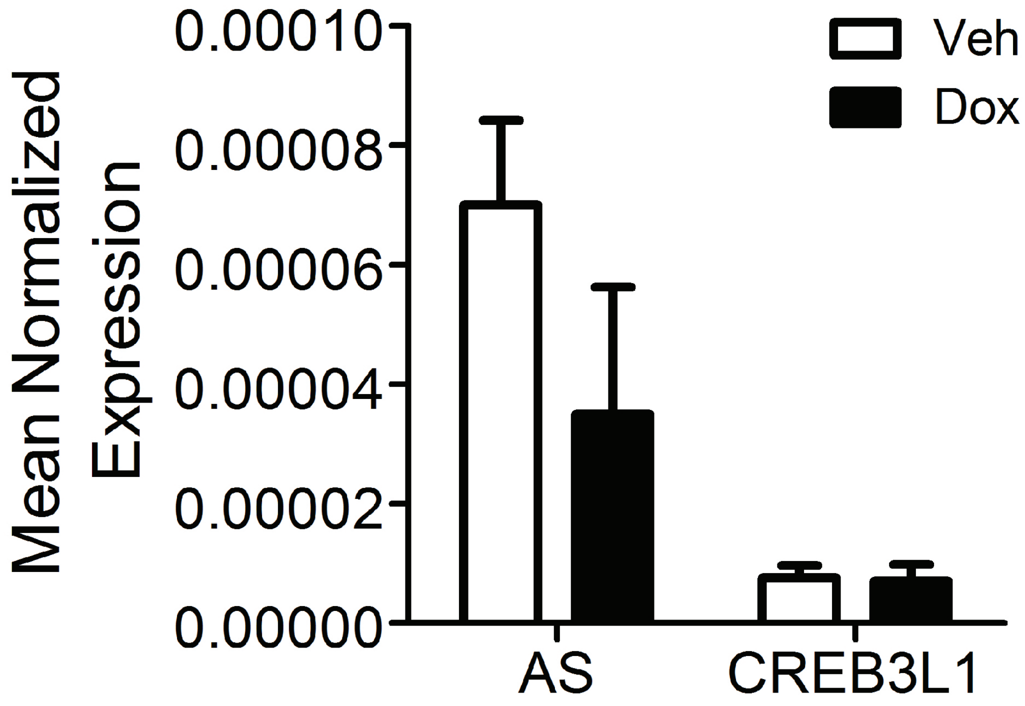

SUPPLEMETARY FIGURE 3: Verification of CREB3L1 knockdown

MCF7 cells were seeded in 60 mm dishes, and siRNA knockdown was performed using AS or CREB3L1 for 24 hours. After that, vehicle or Doxorubicin were added, cells were collected, RNA was isolated and transformed to cDNA and qRT-PCR was performed for CREB3L1, $*p<0.05$ $**p<0.01$.

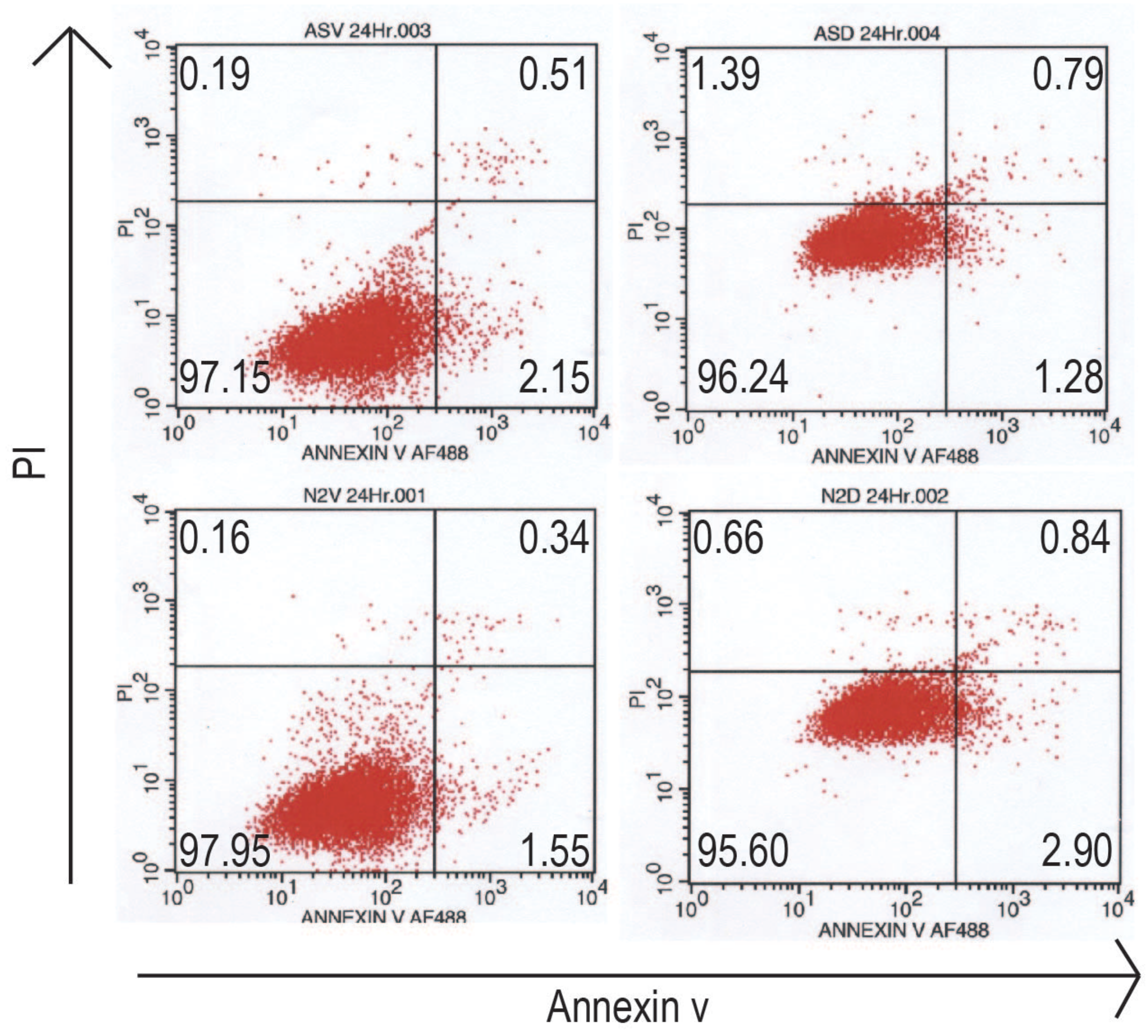

SUPPLEMETARY FIGURE 3: Annexin V/PI staining of cells treated with doxorubicin

MCF7 cells were seeded in 60 mm dishes and siRNA knockdown was performed using AS or nSMase2 (N2) for 24 hours. After which, cells were either treated with vehicle (V) or doxorubicin (D), collected for flow cytometry and stained with annexin V and propidium iodide (PI).
